# Continuity of care in lumbar disc herniation: a systematic review and meta-analysis providing a deeper look into postoperative efficacy

**DOI:** 10.3389/fmed.2025.1536391

**Published:** 2025-07-17

**Authors:** Ying Lin, Qiaoling Chen, RouMei Wang, Bing Zhang, Rui Huang, Yiguang Bai

**Affiliations:** ^1^Department of Orthopedics, Beijing Anzhen Nanchong Hospital, Capital Medical University (Nanchong Central Hospital), Nanchong, China; ^2^Department of Orthopedics, Nanchong Central Hospital, The Second Clinical Medical College of North Sichuan Medical College, Nanchong, China; ^3^Department of Oncology, Beijing Anzhen Nanchong Hospital, Capital Medical University (Nanchong Central Hospital), Nanchong, China; ^4^Department of Oncology, Nanchong Central Hospital, The Second Clinical Medical College of North Sichuan Medical College, Nanchong, China; ^5^Department of Medical Ultrasound, The First Affiliated Hospital of Guangxi Medical University, Nanning, China; ^6^Department of Orthopedics, The Fifth Affiliated Hospital of Guangxi Medical University, Nanning, Guangxi, China

**Keywords:** continuity of care, lumbar disk herniation, meta-analysis, postoperative efficacy, rehabilitation

## Abstract

**Background:**

Lumbar disk herniation is a prevalent spinal disorder. Continuity of care ensures seamless, coordinated, and high-quality management across healthcare transitions to optimize outcomes and reduce readmission risk. Insufficient structured postoperative care programs after diskharge may hinder recovery, lower quality of life, and increase complication risks.

**Objectives:**

This study aims to explore the significance of continuity of care in enhancing the recovery outcomes of patients with lumbar disk herniation post-surgery through a meta-analysis.

**Methods:**

Following the Preferred Reporting Items for Systematic Reviews and Meta-Analyses (PRISMA) guidelines, we conducted an extensive search across several databases, including MEDLINE (PubMed), EMBASE, Web of Science, Google Scholar, CINAHL, EBSCO, Cochrane Library and Scopus, without restrictions on language or publication date. A meta-analysis was performed using both fixed-effects and random-effects models based on heterogeneity assessment. We analyzed the data using R software version 4.2.2 and Stata software version 15.1. The risk of bias was assessed using the Cochrane Risk of Bias Tool, the Newcastle-Ottawa Scale (NOS), funnel plots, sensitivity analysis, Egger’s test, and Begg’s test.

**Results:**

This study included a total of 15 studies, involving 1,804 participants. The meta-analysis results showed that the experimental group had significantly lower Visual Analog Scale (VAS), Oswestry Disability Index (ODI), and Self-Rating Anxiety Scale (SAS) scores 3 months post-operation compared to the conventional control group [SMD = −0.66, 95% CI (−0.91, −0.41), SMD = −0.91, 95%CI (−1.24, −0.58), SMD = −0.61, 95% CI (−0.91, −0.31), *p* < 0.01]. Additionally, the Japanese Orthopedic Association (JOA) score of the experimental group was significantly higher than that of the conventional control group at 3 months post-operation [SMD = 1.70, 95%CI (1.01, 2.39), *p* < 0.01].

**Conclusion:**

Our meta-analysis indicates that continued care interventions significantly alleviate postoperative pain, enhance lumbar function, reduce anxiety, and improve overall functional recovery in LDH patients. High-quality continued care interventions are critical strategies for enhancing postoperative recovery outcomes. Nonetheless, as the included primary studies predominantly originate from China, further validation of these findings in diverse regions and populations is warranted in future research.

**Systematic review registration:**

PROSPERO https://www.crd.york.ac.uk/prospero/, identifier CRD42024604420.

## 1 Introduction

Lumbar disk herniation (LDH) involves degenerative alterations in the lumbar intervertebral disk, leading to the rupture of the annulus fibrosus and the protrusion or displacement of the nucleus pulposus. This condition primarily manifests as lumbar and leg pain due to the irritation or compression of the associated nerves (1). The core strategy for addressing lumbar disk herniation that results in low back and leg pain is to undertake surgical treatment only when conservative therapies have not been effective (2). While surgery can provide short-term pain relief and improve physical function, its long-term effectiveness remains a subject of debate (3). The long-term complications of lumbar disk herniation surgery primarily include spinal instability, adhesions and scar formation, recurrence, and reherniation. In severe cases, these complications can lead to sensory and motor dysfunction in the lower limbs (4–7). Therefore, it is crucial to promote functional recovery, alleviate pain, and minimize postoperative complications following lumbar disk herniation surgery.

However, patients following LDH surgery often struggle to obtain timely professional guidance after discharge, have difficulty accurately understanding medical instructions, and lack knowledge regarding rehabilitation. Continuing care can effectively provide guidance and support to patients during the postoperative recovery process, helping them to address challenges after discharge. Continuing care typically involves the process of moving from a hospital setting back to home, encompassing discharge planning, referrals, and ongoing support and guidance once patients return to their families or communities. This approach addresses the gap in nursing interventions between hospital care and home care (8). Apart from the conventional post-discharge continuing care model, several studies have innovatively utilized internet-based information technologies to develop platforms for ongoing care, benefiting patients who have undergone treatment (9–11). Numerous studies indicate that implementing continuous care for patients after discharge can enhance their recovery ability, improve their quality of life, and reduce readmission rates (12–14).

Currently, there is no systematic and comprehensive evidence-based proof regarding the postoperative efficacy of continued care for LDH patients. Therefore, this study aims to assess the impact of continued care during the 3 months postoperative follow-up on the rehabilitation outcomes of patients with LDH through a systematic meta-analysis.

## 2 Materials and methods

This was a systematic review and meta-analysis conducted strictly according to the PROSPERO registration protocol (CRD42024604420) and PRISMA guidelines.

## 3 Literature search strategy

According to the PICOS framework, the study population includes: (P) patients undergoing surgery for lumbar disk herniation; (I) an intervention involving continued care strategies post-discharge; (C) control patients receiving only traditional discharge care strategies; (O) post-operative clinical assessments and relevant outcome indicators; (S) study types consisting of both randomized and non-randomized controlled trials. We conducted a comprehensive and systematic search across five electronic databases—MEDLINE (PubMed), EMBASE, Web of Science, Google Scholar, CINAHL, EBSCO, Cochrane Library and Scopus—to identify pertinent studies. The search period covered the inception of each database through 1 October 2024. Following the PICOS principles, we developed a systematic search strategy, with detailed search terms and methodology provided in Supplementary Table 1. No language restrictions were applied.

## 4 Inclusion and exclusion criteria

### 4.1 Inclusion criteria

1. Patients who underwent surgery for a lumbar disk herniation and received ongoing care intervention strategies post-discharge;
2. Any study that includes a follow-up period of at least 3 months;
3. The outcome indicators should include at least one of the following: Visual Analog Scale (VAS), Oswestry Disability Index (ODI), Japanese Orthopedic Association Scores (JOA), or Self-Rating Anxiety Scale (SAS), which are commonly used to assess pain, functional recovery, and psychological status in lumbar disk herniation patients and have good comparability in related studies.
4. If the language is Chinese, only articles from core Chinese journals (such as Chinese Social Sciences Citation Index, Chinese Science and Technology Paper and Citation Database, etc.) will be included, or those unanimously assessed as high-quality by three reviewers.

### 4.2 Exclusion criteria

1. Studies with either incomplete or inaccessible data;
2. Studies with a follow-up period of less than three months or not including three months.
3. Animal studies, conference abstracts, case reports, protocols, correspondences, meta-analyses, and other articles.
4. Articles that are not core Chinese journals or cannot be included after evaluation by three reviewers (if written in Chinese).

## 5 Study selection and data extraction

**FIGURE 1:**
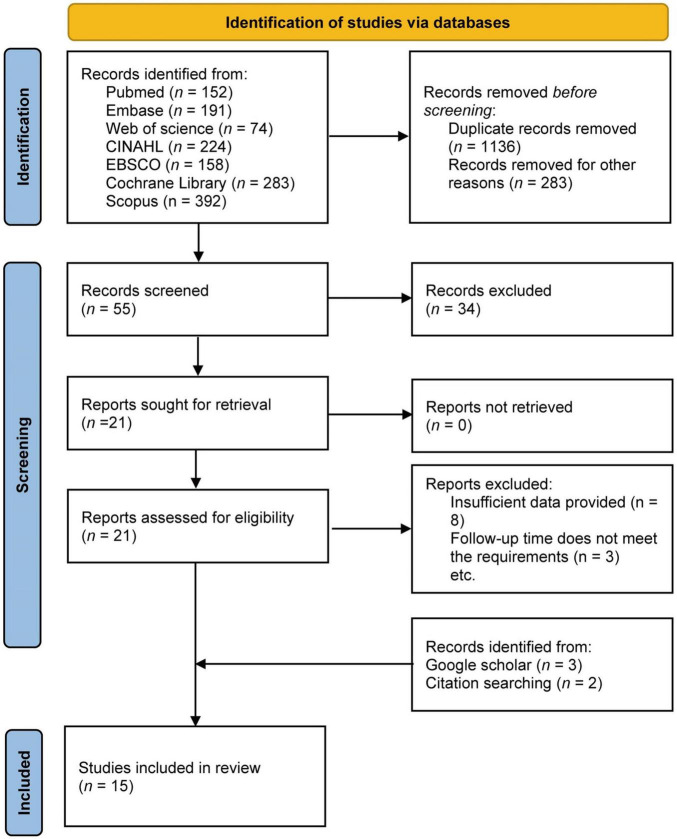
Prisma flow diagram for inclusion and exclusion of studies.

Literature search records were systematically organized using the EndNote 20 software. The selection process was broken down into three distinct stages. For the first stage, a trio of independent reviewers carried out an initial scan based on article titles, retaining those meriting further scrutiny for abstract review. In the second stage, two independent spine orthopedic attending physicians with over 5 years of clinical experience conducted abstract reviews of preliminary selections to assess relevance and suitability. Conflicting opinions were resolved through constructive discussions between the reviewers or, if needed, consultation with another team member was pursued. During the final stage, the same pair of reviewers meticulously examined the full texts of the remaining articles against predetermined inclusion criteria. Any irreconcilable disagreements at this junction were addressed via extensive consultations with the broader review team. The data harvested from these selected studies comprises: (1) author, (2) country, (3) publication year, (4) sample size, (5) sex, (6) age, (7) intervention, and (8) study findings pertaining to the VAS, ODI, JOA, and SAS scores. The literature screening flow chart is depicted in Figure 1.

## 6 Quality assessment and risk of bias assessment

**FIGURE 2:**
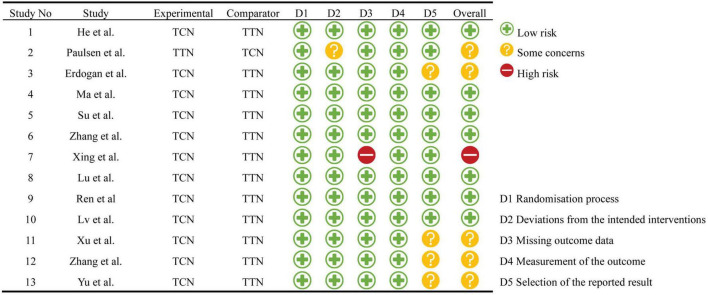
Quality assessment of randomized clinical trials using the Cochrane Risk of Bias tool. TCN, types of continuous nursing; TTN, types of traditional nursing.

The quality and risk of bias in the Randomized Controlled Trials (RCTs) included in this study were rigorously evaluated using the Cochrane Collaboration tool. This tool was applied independently and blindly, with any disagreements that arose being resolved through collective discussion. The evaluation focused on several aspects, specifically the generation of random allocation methods, concealment of the allocation scheme, blinding of patients and staff, blinding of outcome assessors, data comprehensiveness, presence of selective reporting, and potential for other biases. Based on these biases, three categories were identified using the normative standards and assessment criteria of the risk assessment tool: uncertain risk of bias, low risk of bias, and high risk of bias (Figure 2). For non-randomized controlled studies, the Newcastle-Ottawa Scale (NOS) was deployed as a systematic review tool for non-randomized studies, evaluating selection, comparability, and exposure. Every study received a total score out of nine, with a final result of six or more indicating a high-quality document. Any documents scoring under six were deemed low quality (Supplementary Table 2). Lastly, studies requiring ethical approval were to specify both the authorized ethics review agency and the ethics review number.

## 7 Data analysis

The statistical analysis for this study was conducted using R language version 4.2.2, utilizing both the “meta” and “metafor” packages, along with Stata software version 15.1 (Stata Corp LLC, College Station, TX, United States). The outcome of the effect index analysis was denoted by the Standard Mean Difference (SMD) and a 95% confidence interval (CI). A forest plot was created to visually represent the statistical results, using an α = 0.05 as the test standard. Heterogeneity across the results was evaluated via *I*^2^ statistics. In cases where the heterogeneity was low (*I*^2^ < 50%), a fixed-effect model was utilized for further analysis. Conversely, a high heterogeneity (*I*^2^ > 50%) warranted the use of a random effect model, evaluation of sensitivity, and discussion on the source of the heterogeneity. Possible publication biases were assessed through funnel plots, Egger’s test, and Begg’s test. An asymmetrical funnel plot suggested publication bias, which was then quantified utilizing Egger’s and Begg’s test. If the resultant value from either test exceeded 0.05, it was inferred that there was no publication bias present (Supplementary Table 3) (15).

## 8 Results

### 8.1 Characteristics of the included studies

A total of 1,474 records were retrieved through database searches. After excluding 1,136 duplicate studies and 283 articles that did not align with the research focus, we assessed the remaining 55 studies. Upon careful review of the titles and abstracts, 34 articles did not meet the inclusion criteria, eight were excluded due to insufficient data, and three were omitted due to a follow-up duration of less than 3 months. By supplementing the search with manual exploration on Google Scholar and literature tracking, five additional studies were included. A thorough reading of the full texts and comparison with inclusion criteria resulted in a final total of 15 studies being incorporated (16–30) (Figure 1), with the basic demographics listed in Table 1. The studies included in this meta-analysis were published from 2017 to 2024, involving 1,804 participants—comprising 893 in the experimental groups and 911 in the control groups (Table 1). Geographically, one study was conducted in Denmark (22), one in Turkey (16), and 13 in China (17–21, 23–30). In terms of study type, 13 were randomized controlled trials, with seven classified as low risk of bias, one as high risk, and five as moderate concern (Figure 2). For the two non-randomized controlled trials, we employed the NOS for quality assessment, with Li et al. rated as “High quality” and Wu et al. as “Medium quality.” Among the included studies, interventions varied: six studies (17, 18, 20, 25, 28, 30) used WeChat group supervision on top of traditional care, Erdogan et al. (16) implemented computer-assisted training, Ma et al. (21) employed home or community rehabilitation care, Su et al. (24) utilized APP continuous nursing, Zhang et al. (29) adopted seamless family continuous care, Xing et al. (26) used internet interactive measures, Lu et al. (19) employed a responsibility system of continuous nursing, Ren (23) applied hospital-community home care, and Xu et al. (27) used health education videos. The corresponding control groups received only traditional discharge care, with a unique case by Paulsen et al. (22) defining the research group as standard care and the control group as patients receiving municipal rehabilitation treatment 4–6 weeks post-lumbar disk herniation surgery. Follow-up durations ranged from 1 to 24 months, with the most common being 3 months. The main outcome measures included VAS, ODI, JOA, and SAS. Of these, six articles reported outcomes related to VAS score for back pain 3 months post-LDH surgery; after excluding one study due to high heterogeneity, five articles were included in the analysis. Furthermore, 13 studies reported outcomes related to ODI scores 3 months post-LDH surgery; after removing two due to high heterogeneity and one focused on ODI scores 12 months post-surgery, 10 studies were incorporated in the statistical analysis. Additionally, eight articles reported JOA scores at the 3 months follow-up, and two studies reported SAS scores at the same interval. Given the variation in follow-up durations across studies, but with all included studies reporting 3 months postoperative outcomes, we prioritized this time point for analysis—a choice aligned with clinical practice and consistent with established standards for assessing early recovery and efficacy in previous research (31). Due to limited study numbers, inconsistency in measurement units, or inadequate data provision, other outcome measures such as MBI and SDS were subject to descriptive analysis.

**TABLE 1:** Characteristics of the studies included in the network meta-analysis.

Study	Year	Country	Studies types	sample size	Sex (female/male) (S/C)	Age (mean ± SD) (S/C)	Study group interventions	Duration of follow-up	Main outcome measures
Li et al.	2024	China	Retrospective case control	277	45: 87/58: 87	42.1 ± 12.76/44.4 ± 16.86	Traditional discharge nursing + wechat group supervision	3 and 12 months after Surgery	a–b
Xing et al.	2022	China	Randomized controlled trial	86	16: 27/22: 21	NA	Routine rehabilitation guidance + internet interactive follow-up	Before surgery, 3 days, 3 months, and 6 months after surgery	a–d, g–h
Zhang et al.	2021	China	Randomized controlled trial	80	19: 21/22: 18	57.6 ± 3.4/58.9 ± 2 4	Traditional diskharge nursing + wechat group supervision	At discharge, 2 and 3 months after discharge	b
Zhang et al.	2020	China	Randomized controlled trial	64	16: 16/15: 17	46.23 ± 4.98/47.75 ± 5.13	Traditional discharge nursing + seamless family continuous care	Pre-intervention, 1 and 3 months intervention	b–c
He et al.	2020	China	Randomized controlled trial	95	22: 25/21: 27	46.01 ± 5.12/45.88 ± 4.99	Traditional discharge nursing + wechat group supervision	After 3 months of follow-up	b–c, e
Erdogan et al.	2020	Turkey	Randomized controlled trial	62	14: 17/13: 18	39.97 ± 10.89/40.58 ± 11.45	Computer-assisted training	15 days, 1 and 3 months after surgery	b, f
Lv et al.	2019	China	Randomized controlled trial	84	19: 23/16: 26	55.68 ± 7.67/55.36 ± 7.42	Traditional discharge nursing + wechat group supervision	At discharge and 3 months after surgery	b–c
Yu et al.	2019	China	Randomized controlled trial	100	13: 37/12: 38	45.71 ± 10.75/46.19 ± 10.53	Traditional discharge nursing + wechat group supervision	3, 6 months after discharge	a–d
Paulsen et al.	2019	Denmark	Randomized controlled trial	146*	27: 46/27: 46	42.80 ± 11.80/42.90 ± 8.90	Standard of care	After 1, 3, 6, 12, 24 months of follow-up	a–b, i
Lu et al.	2018	China	Randomized controlled trial	80	11: 29/14: 26	57.3 ± 3.6/58.5 ± 5.4	Traditional discharge nursing + responsibility system continuous nursing	1, 4, 12 weeks after discharge	a–c
Xu et al.	2018	China	Randomized controlled trial	167	31: 51/40: 45	25.38 ± 7.14/24.58 ± 8.35	Traditional discharge nursing + health education video	Before surgery, 1 and 3 months after surgery	b
Ren et al.	2018	China	Randomized controlled trial	200	45: 55/39: 61	56.80 ± 2.31/55.47 ± 6.43	Traditional discharge nursing + hospital-community home care	Traditional discharge nursing	b–c
Ma et al.	2017	China	Randomized controlled trial	115	25: 32/24: 34	57.30 ± 6.45/56.07 ± 7.58	Home or community rehabilitation care	Before surgery, 1 and 3 months after surgery	b–c
Wu et al.	2017	China	Case control	80	8: 32/10: 30	45.6 ± 11.1/44.9 ± 11.2	Traditional discharge nursing + wechat group supervision	After 3 months of follow-up	a, d
Su et al.	2017	China	Randomized controlled trial	168	36: 48/38: 46	32.62 ± 9.61/32.80 ± 9.34	Traditional discharge nursing + APP continuous nursing	Before and 3 months after surgery	b–c

^a^VAS, Visual Analog Scale. ^b^ODI, Oswestry disability index. ^c^JOA, Japanese Orthopedic Association scores. ^d^SAS, Self-Rating Anxiety Scale. ^e^36-item short form health survey. ^f^STAI, State-Trait Anxiety Inventory. ^g^MBI, modified Barthel Index. ^h^SDS, Self-Rating Depression Scale. ^i^EQ5D, Euro Quality of life five-dimensional questionnaire. *Three months post-surgery, 141 participants were followed up (72 in the study group, 69 in the control group).

### 8.2 Meta-analysis of the principal outcome

#### 8.2.1 Visual analog scale score (VAS)

**FIGURE 3:**
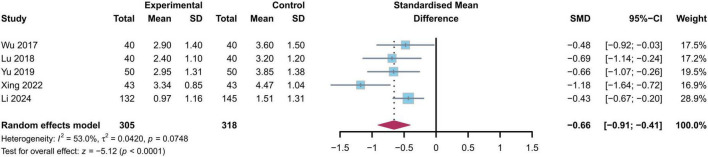
Forest plot comparing changes in VAS pain scores between the study group and control group at 3 months postoperative follow-up. VAS, Visual Analog Scale, SMD, standardized mean difference.

Among the numerous tools used for pain assessment, the VAS is a reliable instrument for evaluating the severity of pain. Out of six studies reporting outcomes related to VAS back pain scores, one study with high heterogeneity was excluded via sensitivity analysis, resulting in a total of five studies being included in the final analysis (18, 19, 25, 26, 28). The experimental group comprised 305 subjects, while the control group consisted of 318 subjects (Figure 3). Heterogeneity testing revealed some degree of disparity among studies (*I*^2^ = 53%, *p* = 0.07), thus a random-effects model was employed for analysis. The results indicated that the experimental group showed a significant reduction in VAS back pain scores at three months post-operatively when compared to the standard control group [SMD = −0.66, 95% CI (−0.91, −0.41), *p* < 0.01].

#### 8.2.2 Oswestry disability index (ODI)

**FIGURE 4:**
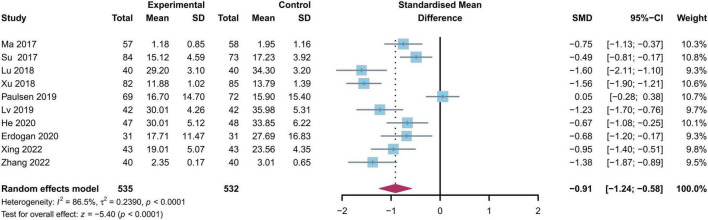
Forest plot depicting the comparison of ODI Score changes between the study and control groups at the 3 months postoperative follow-Up. ODI, Oswestry disability index; SMD, standardized mean difference.

The ODI is another frequently used scoring system for patients suffering from lumbar disk herniation. Through sensitivity analysis, we eliminated two studies with high heterogeneity and one study whose result was the ODI score at the 12 months follow-up after LDH surgery. Ultimately, there were ten studies reporting on ODI scores, encompassing 535 cases in the experimental group and 532 cases in the control group (Figure 4) (16, 17, 19–24, 26, 27, 29, 30). The heterogeneity test revealed substantial heterogeneity among the studies (*I*^2^ = 86.5%, *p* < 0.01), thus a random effects model was selected for analysis. The analysis indicated that the ODI score of the experimental group was significantly lower than that of the regular control group three months after the surgery [SMD = −0.91, 95%CI (−1.24, − 0.58), *p* < 0.01].

#### 8.2.3 Japanese Orthopedic Association Scores (JOA)

**FIGURE 5:**
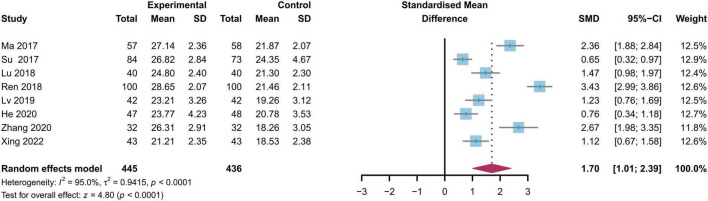
Forest plot comparison of postoperative JOA Score changes between study and control groups at 3 months follow-up. JOA, Japanese Orthopedic Association; SMD, standardized mean difference.

The JOA assessment treatment scores, are primarily used to evaluate physical disabilities. Eight studies reported results regarding JOA scores, involving 445 cases in the experiment group and 436 cases in the control group (Figure 5) (17, 19–21, 23, 24, 26, 29). Heterogeneity testing showed considerable variability among the studies (*I*^2^ = 95%, *p* < 0.01), prompting us to choose a random-effects model for analysis. The analysis demonstrated that the JOA scores of the experimental group were significantly higher than those of the routine control group three months post-operation [SMD = 1.70, 95%CI (1.01, 2.39), *p* < 0.01].

#### 8.2.4 Self-Rating Anxiety Scale (SAS)

**FIGURE 6:**
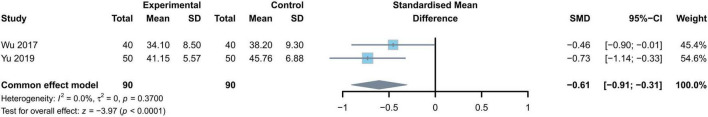
Forest plot analysis of changes in SAS scores between study and control groups at 3 months postoperative follow-up. SAS, Self-Rating Anxiety Scale; SMD, standardized mean difference.

The SAS is recognized as a standardized inventory for gauging anxiety. It measures the severity of anxiety symptoms and the changes that may occur during treatment. Two studies reported outcomes pertaining to SAS scores, including 90 subjects in the experimental group and 90 in the control group (Figure 6) (25, 28). Heterogeneity tests exhibited low inconsistency across studies (*I*^2^ = 0%, *p* = 0.37), which led us to opt for a fixed-effects model in our analysis. The findings suggest that the experimental group had significantly lower SAS scores three months postoperatively compared to the standard control group [SMD = −0.61, 95% CI (−0.91, −0.31), *p* < 0.01].

#### 8.2.5 Other results

In addition to the primary outcomes, the studies we included reported on additional indicators related to the efficacy of continued care following surgery for lumbar disk herniation. He et al. (17) reported that continuity of care based on the WeChat platform can significantly improve the quality of life for patients undergoing LDH surgery. Paulsen et al. (22) statistical analysis revealed no difference between the groups in terms of satisfaction in either the intent-to-treat, as-treated or per protocol analysis. The continued care group exhibited improvements in rehabilitation completion, efficacy satisfaction, and re-herniation worry scores [Li et al. (18); Table 2], level of information, STAI [Erdogan et al. (16)], Medication compliance [Wu et al. (25); Table 2], clinical nursing outcomes [Ma et al. (21), Ren et al. (23)], adherence to functional exercise [Su et al. (24), LV et al. (20)] and satisfaction rates [Su et al. (24)], Excellent and good rate of rehabilitation effect [Zhang et al. (29)], Health Behavior Score [Lu et al. (19); Table 2], lumbar spine joint mobility, MBI, anxiety and depression conditions 6 months post-operation [Xing et al. (26), Table 2], readiness for discharge, neurological function recovery [Xu et al. (27); Table 2], Pain degree after nursing, excellent and good rate of compliance [Zhang et al. (30)], Compliance and Rehabilitation Effects [Yu et al. (28)] also showed significant improvement in the continued care group compared to the control group (*p* < 0.05).

**TABLE 2:** Additional outcomes related to the transitional care strategy.

Study	Indicators	Study group	Control group	*P*
Li et al.^c^	Rehabilitation completion score	7.98 ± 1.857	4.14 ± 3.066	<0.001
	Efficacy satisfaction score	9.13 ± 1.101	8.28 ± 1.240	<0.001
	Re-herniation worry score	1.97 ± 1.217	2.79 ± 1.973	<0.001
Erdogan et al.^a^	Information scores	46.74 ± 2.19	41.61 ± 4.54	0.001
	State anxiety	30.55 ± 6.75	42.65 ± 12.16	0.001
	Trait anxiety	37.29 ± 7.25	43.94 ± 6.91	0.001
Wu et al.^c^	Medication compliance	3.6 ± 0.7	3.3 ± 0.5	0.030
Lu et al.^c^	Health behavior score	49.6 ± 3.8	39.4 ± 3.3	<0.001
Xing et al.	MBI^a^	72.21 ± 11.35	65.53 ± 12.38	<0.01
	MBI^b^	86.03 ± 11.41	79.41 ± 10.32	<0.05
	Lumbar forward flexion^b^	42.54 ± 6.42	38.31 ± 5.23	<0.01
	Lumbar spine extension^b^	20.66 ± 3.54	17.83 ± 3.01	<0.01
	SDS^b^	43.64 ± 4.88	48.36 ± 4.96	<0.05
	SAS^b^	42.68 ± 4.65	48.65 ± 5.63	<0.05
Xu et al.^a^	Readiness for hospital discharge	162.39 ± 5.55	155.95 ± 10.27	<0.01

^a^Is 3 months post-surgery. ^b^Is 6 months post-surgery. ^c^Is 12 months post-surgery. STAI, State-Trait Anxiety Inventory; MBI, modified Barthel Index; SDS, Self-Rating Depression Scale; SAS, Self-Rating Anxiety Scale.

## 9 Publication bias

**FIGURE 7:**
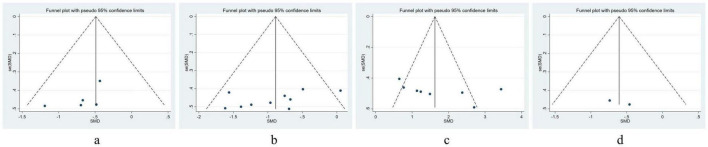
Funnel plot of publication bias. **(a)** VAS, Visual Analog Scale; **(b)** ODI, Oswestry disability index; **(c)** JOA: Japanese Orthopedic Association Scores; **(d)** SAS, Self-Rating Anxiety Scale.

By utilizing a funnel plot, we evaluated the presence of publication bias across the incorporated studies and based on Figure 7, the funnel plot did not significantly illustrate publication bias. Furthermore, we evaluated the risk of bias for each included article, employing Begg’s and Egger’s regression tests, which according to Supplementary Table 3, suggested no significant bias in the results of the meta-analyses.

## 10 Discussion

Early postoperative care guidance following LDH surgery can facilitate the recovery of lumbar and lower limb muscle function (32). Additionally, engaging in long-term functional exercise post-surgery helps modulate muscle tension and enhance muscle strength, effectively preventing the recurrence of LDH and improving patients’ quality of life. The extent to which patients continue functional exercise after discharge significantly impacts their treatment outcomes and quality of life. However, many patients currently face challenges such as insufficient intensity, low frequency, and short duration in their exercise routines. Thus, healthcare workers need to implement continuous care interventions (20). This study is the first to examine the impact of extended nursing on postoperative outcomes in patients with lumbar disk herniation through the lens of a systematic review and meta-analysis.

Fifteen studies involving 1,804 participants were included in our analysis. The results indicated that continuous care was more effective than the control group in alleviating postoperative pain in patients with LDH. The treatment goals for LDH include pain relief, improved functionality, and enhanced quality of life. For most patients with significant neurological symptoms, such as radicular pain, discectomy can alleviate symptoms of disk herniation (33). However, a systematic review revealed that 25% of patients might experience recurrent back pain 2 years after undergoing surgery for LDH (34). Pain-related distress can hinder physical activity, severely affect a patient’s normal work and life, and increase psychological burdens. The concept of “post-surgical spine syndrome” has recently been proposed, characterized as chronic or newly developed pain following spinal surgery, potentially associated with nerve root damage, compression, arachnoiditis, epidural fibrosis, adjacent segment degeneration, and spinal instability (35). Early rehabilitation can help prevent the degeneration of lumbar muscles such as the multifidus and erector spinae, enhancing the stability of the lumbar spine (36). Therefore, we believe that alleviating postoperative pain in LDH patients should be a primary goal of continuous care interventions. Li et al. (18) conducted a long-term follow-up of 282 patients undergoing UBED surgery using an internet platform and found that continuous care programs could alleviate both short-term and long-term persistent lumbar pain symptoms in these patients. The benefits of continuous care are not limited to postoperative spinal patients. for instance, Elahi et al. (37) reported that a continuous care model effectively reduced pain symptoms in breast cancer chemotherapy patients. Another study indicated that implementing a continuous care plan could significantly reduce the frequency of chest pain episodes in myocardial infarction patients. Continuous care is a cost-effective, non-pharmacological method (38).

After orthopedic surgery, the healing process for muscles, ligaments, and bones is relatively long. If rehabilitation training is not conducted in a timely manner, it may lead to poor prognosis or symptoms such as joint stiffness and functional impairment (39). Our study indicated that the ODI scores of the continuation care group were significantly lower, and the JOA scores were significantly higher than those of the control group. A systematic review and meta-analysis by Özden et al. (40) reported that early rehabilitation following lumbar surgery effectively alleviates functional disorders. Within the studies we examined, He et al. (17) followed 95 patients who underwent LDH surgery and found notable improvements in ODI and JOA scores in the continuation care group. It is reported that postoperative rehabilitation, disk herniation type, and age are independent risk factors affecting the prognosis after lumbar intervertebral disk herniation surgery (32). Schwartz et al. (41) conducted a prospective cohort study, concluding that exercise post-spinal surgery enhances psychological health and spinal recovery, recommending long-term functional exercises. These findings align with our results and further validate the acknowledgment of postoperative lumbar disk herniation rehabilitation by numerous scholars (32, 42, 43). Another critical factor to consider is the SAS, as psychological disorders and anxiety are common among LDH patients post-surgery, markedly affecting their recovery process. For instance, a study found that patients contracting COVID-19 showed increased postoperative anxiety following LDH surgery, demonstrating a case involving comorbidities (44). Even in the absence of complications, chronic pain caused by LDH can be an important factor in causing psychological disorders. Cai et al. (45), using an LDH animal model, observed elevated TNF-α mRNA expression and reduced 5-HT levels in rats experiencing chronic pain, indicating that LDH-induced pain can trigger depressive behaviors. Our meta-analysis further demonstrated that continuity of care significantly reduced SAS scores, emphasizing the need for structured follow-up and psychological support in postoperative management. The improvement in both psychological and functional outcomes with continuity of care may be explained by several theoretical mechanisms. Firstly, continuous care interventions provide stable psychological support, enhancing patients’ sense of security and trust, which effectively reduces anxiety levels and promotes recovery (46). This mechanism may be related to the role of interventions that offer social support and emotional comfort. Secondly, patients’ trust in and adherence to the rehabilitation process are key factors in treatment outcomes. Continuous care improves patients’ sense of self-efficacy and engagement, thereby accelerating recovery. Furthermore, ongoing education and psychological support can enhance patients’ knowledge of recovery and foster positive emotions, further improving postoperative function (47). Therefore, continuity of care not only directly contributes to medical interventions but also strengthens psychological support and recovery beliefs, thereby enhancing overall recovery outcomes. Therefore, beyond promoting rehabilitation exercises, addressing psychological and social barriers is essential for optimizing recovery.

Continuity of care is a core dimension of high-quality care in the management of disease (3). Traditionally, patients recovering from lumbar surgery prefer to recuperate at home, depending on outpatient follow-ups for wound healing, symptom monitoring, and disease progression. It is noteworthy that post-operative recovery is a long-term process (18). This study aims to systematically evaluate and meta-analyze the impact of continuity of care on post-operative outcomes in patients with LDH. The findings demonstrate that at three months post-operation, the experimental group had significantly lower VAS, ODI, and SAS scores, and notably higher Japanese JOA scores compared to the control group. These outcomes hold clinical significance, highlighting quality continuity care interventions as a vital strategy for enhancing post-operative recovery. However, the study has certain limitations. Firstly, selection bias may affect our results, as there are potential differences in the inclusion criteria, patient characteristics, and healthcare settings across the studies we included, which may limit the comparability between them. Despite strictly adhering to the inclusion and exclusion criteria, the number of eligible studies remains limited, and some relevant studies may have been overlooked, thus affecting the representativeness of our findings. Therefore, larger-scale studies or meta-analyses are needed in the future to validate and strengthen our conclusions. Secondly, alongside randomized controlled trials, we included retrospective and case-control studies, which may impact overall result predictions. Additionally, as most of the studies originated from China, differences in healthcare systems, treatment protocols, and cultural backgrounds across countries, coupled with variations in genetic and demographic characteristics of the patient populations, may influence recovery outcomes. These factors suggest that the findings of this meta-analysis may not be fully generalizable to other regions or populations. Therefore, future research should include diverse populations from different countries and regions to validate these results and assess the impact of regional healthcare practices and genetic factors on postoperative recovery. Future research should encompass studies from more diverse regions and countries. Furthermore, some heterogeneity is observed in the meta-analysis results, potentially due to the inherent study variations or confounding factors. Although most studies control for confounders, it remains uncertain if these adjustments are comprehensive. For instance, patients might seek care from different institutions and consult multiple specialists, potentially receiving conflicting advice, leading to diminished continuity of care quality or affecting feedback outcomes (48). Additionally, the pain score (VAS) exhibited moderate heterogeneity, suggesting that while differences in follow-up methods and nursing details exist among studies, the effect of continuity of care in alleviating postoperative pain remains relatively consistent. However, functional recovery indicators (ODI and JOA) demonstrated high heterogeneity, which may be attributed to variations in patient age, disease severity, surgical approaches, rehabilitation strategies, patient adherence, and healthcare systems. Additionally, the anxiety score (SAS) showed low heterogeneity, indicating that continuity of care has a stable effect in reducing postoperative anxiety. This suggests that standardized postoperative follow-up and psychological support may have broad applicability in anxiety management. However, given the limited number of included studies, further research is needed to strengthen this conclusion. For outcomes with high heterogeneity, the small number of eligible studies prevented us from conducting a more detailed subgroup analysis. Despite some limitations, this study reached a unified conclusion based on high-quality original research, which has important clinical significance. Therefore, future studies should focus on high-quality, standardized research to further validate the clinical value of continuity of care and optimize postoperative rehabilitation strategies. This meta-analysis highlights the essential role of continuity of care in postoperative rehabilitation for lumbar disk herniation. The findings indicate that integrating systematic rehabilitation programs into routine postoperative care can effectively enhance functional recovery and optimize the rehabilitation process. Given the long-term impact of surgical complications, clinicians should consider adopting personalized or information technology-assisted continuity of care models to improve patient outcomes. Future researchers could explore the use of artificial intelligence to further support continuity of care strategies and analyze the impact of these interventions (49). Future clinical guidelines should incorporate evidence-based rehabilitation strategies to ensure comprehensive post-discharge management.

## 11 Conclusion

Our meta-analysis indicates that continued care interventions significantly alleviate postoperative pain, enhance lumbar function, reduce anxiety, and improve overall functional recovery in LDH patients. High-quality continued care interventions are of substantial value in optimizing postoperative recovery outcomes. However, given that the primary studies included in this analysis are largely from China, it is essential to validate these findings in different regions and populations in future studies.

## Data Availability

The raw data supporting the conclusions of this article will be made available by the authors, without undue reservation.
