# Supplementary material for: Continuity of care in lumbar disc herniation: a systematic review and meta-analysis providing a deeper look into postoperative efficacy
Source: Front Med (Lausanne). 2025 Jul 17;12:1536391. doi: 10.3389/fmed.2025.1536391 (PMC12310576; doi:10.3389/fmed.2025.1536391)
Supplement: Supplementary file 1 [file Table_1.docx]

Supplementary Material

**Table S1. Literature Search Strategy**

| Block | Search Concept | Search Terms |
| --- | --- | --- |
| Block 1 | Lumbar Disc Herniation & Surgical Treatment | "Lumbar Disc Herniation" OR "Lumbar Disc Disease" OR "Herniated Disc" OR "Herniated Lumbar Disc" OR "LDH" OR "Intervertebral Disc Degeneration" OR "Lumbar Disc Prolapse" OR "Lumbar Disc Extrusion" OR "Discectomy" OR "Lumbar Discectomy" OR "Microdiscectomy" OR "Micro Discectomy" OR "Transforaminal Lumbar Discectomy" |
| Block 2 | Care Interventions | "Continuity of Patient Care" OR "Care Continuity" OR "Patient Care Continuity" OR "Continuity of Care" OR "Care Continuum" OR "Continuum of Care" OR "Nursing Continuity" OR "Continuity of Nursing Care" OR "Nursing Care Continuity" OR "Discharge Planning" OR "Patient Discharge" OR "Discharge, Patient" OR "Discharges, Patient" OR "Patient Discharges" OR "Discharge Plannings" OR "Planning, Discharge" OR "Plannings, Discharge" OR "Postoperative Care" OR "Post-Surgical Care" OR "Surgical Recovery" OR "Postoperative Recovery" OR "Postoperative Management" OR "Rehabilitation Adherence" OR "Rehabilitation Compliance" OR "Adherence to Rehabilitation" OR "Rehabilitation Follow-up" OR "Post-Surgery Rehabilitation" OR "Surgical Rehabilitation" |
| Block 3 | Digital Health & Computer-Based Support | "Internet" OR "Online Support Platform" OR "WeChat" OR "Mobile App" OR "Mobile Applications" OR "mHealth" OR "Telemedicine" OR "eHealth" OR "Digital Health" OR "Health Information Technology" OR "Smartphone Applications" OR "Computer-Based System" OR "Computerized System" OR "Computer-assisted" OR "Computerized Decision Support" OR "Electronic Health Records" OR "Health Information Systems" |

**Table S2. Newcastle-Ottawa scale for quality assessment of case control**

| Study no | Study | Selection | Comparability |  | Exposure | Overall score | Quality of study |
| --- | --- | --- | --- | --- | --- | --- | --- |
| 1 | Li et al. | ★★★ | ★ |  | ★★★ | 7/9 | High |
| 2 | Wu et al. | ★★ | ★ |  | ★★ | 5/9 | Medium |

*High quality: 7–9

*Medium quality: 4–6

**Table S3. Treatment Relative Ranking of Estimated probabilities**

| Indicators | P for Egger’s test | P for Begg’s test |
| --- | --- | --- |
| VAS | 0.334 | 0.060 |
| ODI | 0.126 | 0.152 |
| JOA | 0.174 | 0.063 |
| SAS | - | 0.317 |

**Figure S1 sensitivity analysis**

|  |  |
| --- | --- |
| **Figure S1a VAS** | **Figure S1b ODI** |
|  |  |
|  |  |
| **Figure S1c JOA** | **Figure S1d SAS** |
